# Genome sequence dataset of *Bacillus altitudinis* strain ST14 isolated from Tunggak River in Gebeng Industrial Park, Kuantan, Pahang

**DOI:** 10.1016/j.dib.2022.108718

**Published:** 2022-11-02

**Authors:** Asmadamia Abdul Aziz, Azuan Syafiq Zulbahri, Mohd Faez Sharif, Mohd Azrul Naim Mohamad, Han Min Gan, Nur Hafizah Azizan

**Affiliations:** a Department of Biotechnology, Kulliyyah of Science, International Islamic University Malaysia, 25200 Kuantan, Pahang, Malaysia; b GeneSEQ Sdn Bhd, Bukit Beruntung 48300 Selangor, Malaysia; c Department of Biological Sciences, Sunway University, 47500 Bandar Sunway, Selangor, Malaysia

**Keywords:** Bioremediation, *Bacillus* sp., Draft genome, Bacteria, Hydrocarbon contamination

## Abstract

*Bacillus* sp. has been reported to be involved in the biodegradation of various hydrocarbon pollutants which can potentially be useful in cleaning up hydrocarbon pollutants. Here we report the draft genome of *Bacillus altitudinis* strain ST14 isolated from Tunggak river, Gebeng, Kuantan, an area in close proximity to industrial activities. Genome sequencing was conducted using Illumina NovaSEQ 6000 technology. Structural genes in the genome were described, including rRNAs, tRNAs, and ncRNAs. *Bacillus altitudinis* strain ST14 was sequenced with a length of 3,801,811 bp containing 3,891 coding sequences (CDS). Functional gene annotation reported the presence of six enzymes involved in the degradation of aromatic compounds often found in hydrocarbon pollutants.

**Specifications Table**

**Table:** 

Subject	Biology
Specific subject area	Microbiology, Genomics, Biotechnology
Type of data	Table, Sequence
How the data were acquired	*Bacillus altitudinis* sample was grown overnight in Luria-Bertani broth. Genomic DNA of *Bacillus altitudinis* was extracted using Macherey-Nagel Nucleospin Tissue DNA Extraction Kit. The draft genome sequence was processed using Illumina NovaSEQ 6000 instrument.
Data format	Raw, analyzed, and deposited
Description of data collection	*Bacillus altitudinis* strain ST14 was isolated from water samples obtained from a river in Malaysia. *Bacillus altitudinis* strain ST14 was able to grow in minimal salt medium (MSM) supplemented with 1% engine oil as its sole carbon source. Genomic DNA was extracted from a pure overnight grown culture of *Bacillus altitudinis* strain ST14.
Data source location	*Bacillus altitudinis* strain ST14 was isolated from water samples collected from Tunggak river located adjacent to Gebeng Industrial Park, Kuantan, Pahang, Malaysia, an area in close proximity to industrial activities. Latitude and longitude: between 30° 56′ 06″ to 30° 59′ 44″ N and 1030° 22′ 42″ to 1030° 24′ 47″ E.
Data accessibility	Data are publicly available at NCBI GenBank: https://www.ncbi.nlm.nih.gov/nuccore/JAMJWU000000000.1/ https://www.ncbi.nlm.nih.gov/bioproject/PRJNA812005 https://www.ncbi.nlm.nih.gov/biosample/SAMN26365749/ https://www.ncbi.nlm.nih.gov/sra/SRX14338850

## Value of the Data

• The draft genome sequence of *Bacillus altitudinis* strain ST14 provides an understanding of bacteria isolated from Tunggak river, an area in close proximity to industrial activities.
• The data obtained from the draft genome of *Bacillus altitudinis* strain ST14 can be useful to conduct extensive research on biodegradation study.
• Data on the genome sequence of *Bacillus altitudinis* strain ST14 is useful for researchers to better understand the genetic features of the bacterium and insights into its biodegradation properties.
• Data can be used for comparative genomics, proteomics, and other evolutionary studies with another *Bacillus* sp. involved in biodegradation.

## 1 Objective

This dataset is generated to investigate the presence of genes involved in the degradation of hydrocarbons in the bacterial isolates, *Bacillus altitudinis* strain ST14. This can be useful to determine the ability of this bacterium to degrade hydrocarbon, thus, might be used as a reference to develop an effective strategy for eliminating hydrocarbons from the environment.

## 2 Data Description

**Fig. 1:**
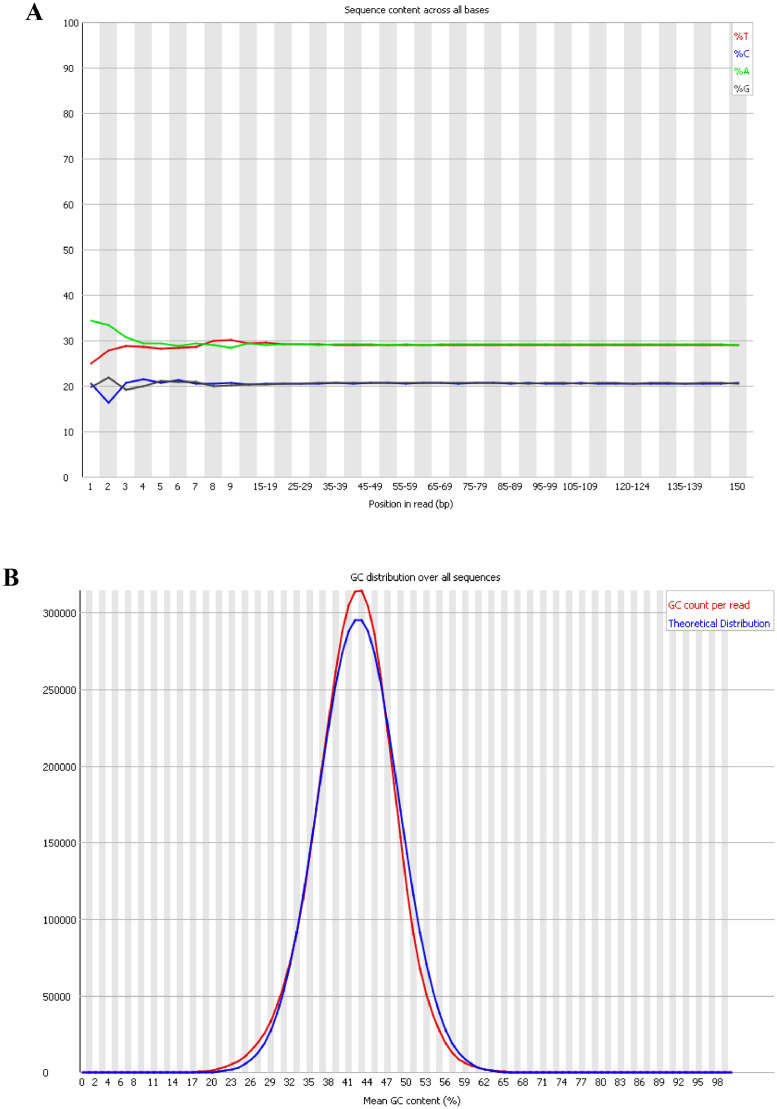
Overview of the *Bacillus altitudinis* strain ST14 raw data quality control assessed using fastQC software. **(A)** Per base sequence content; **(B)** Per base GC content.

The draft genome of *Bacillus altitudinis* strain ST14 was sequenced with a coverage of 100.0X and consisted of 17 contigs with an accumulated length of 3,801,811 bp. The draft genome was assembled with an N_50_ value of 818,136 bp and 41.24% GC content (Table 1). Fig. 1 shows the sequence quality control assessed in the FASTQC format.

**Table 1:** General features of *Bacillus altitudinis* strain ST14.

Attribute	Description
Genome size (bp)	3,801,811 bp
Number of contigs	17
N_50_ (bp)	818,136 bp
G+C content (%)	41.24%
CDS (coding sequences)	3,891
tRNA number	57
rRNA number	5
Non-coding RNA number	5
GenBank accession	JAMJWU000000000
BioSample accession	SAMN26365749
BioProject accession	PRJNA812005

Taxonomic identification of *Bacillus altitudinis* strain ST14 shows the highest Average Nucleotide Pairwise similarity to *Bacillus altitudinis* 41KF2b^T^ (98.39%) and *Bacillus altitudinis* W3 (98.38%) (Table 2).

**Table 2:** Taxonomic identification of *Bacillus altitudinis* strain ST14 based on ANIb.

	B. altitudinis strain ST14	B. altitudinis 41KF2b^T^	B. altitudinis W3
B. altitudinis strain ST14	*	98.23 %	98.38%
B. altitudinis 41KF2b^T^	98.39 %	*	98.35 %
B. altitudinis W3	98.38 %	98.19 %	*

Gene annotation and prediction reveal the presence of six enzymes responsible for aromatic compound degradation in the draft genome, including three alcohol dehydrogenase, one 4-carboxymuconolactone decarboxylase, one 4-oxocrotonate tautomerase, and one catechol 2,3-dioxygenase. The information obtained about the genome sequence of *Bacillus altitudinis* strain ST14 and the diversity of the annotated genes can improve the understanding of this bacterium on aromatic compound degradation.

## 3 Experimental Design, Materials and Methods

Tunggak river is located adjacent to Gebeng Industrial Park Kuantan, an area in close proximity to industrial activities. Aromatic hydrocarbons are ubiquitous anthropogenic pollutants that are commonly related to industrial activities and become a global concern due to their harmful and toxic properties [1]. Prolonged exposure to these pollutants leads to an adverse effect on the environment and human beings which needs to be eliminated [2]. The discovery of indigenous bacteria capable of degrading hydrocarbons offers a promising solution to eliminate these pollutants from the environment [3], [4], [5]. *Bacillus* sp. has been reported to be involved in the biodegradation of various hydrocarbon pollutants which can potentially be useful in cleaning up hydrocarbon pollutants [6,7]. Therefore, the draft genome of *Bacillus altitudinis* strain St14 was reported in this finding.

*Bacillus altitudinis* strain ST14 was isolated from water samples obtained from the Tunggak river that was reported to be polluted with oil contamination [8]. The isolate was grown at 30°C for 20 h on minimal salt medium (MSM) agar enriched with 1% engine oil as the sole carbon source. The obtained colony was streaked on nutrient agar to obtain the pure culture that was able to grow in hydrocarbons. Genomic DNA of *Bacillus altitudinis* strain ST14 was extracted using Nucleospin® Tissue DNA Extraction Kit (Macherey-Nagel, Düren, Germany) from an overnight culture grown in Luria-Bertani broth at 30°C and 150 rpm. The NEB Ultra II DNA Library Prep Kit (NEB, Ipswich, MA) was used to construct the DNA library, and whole-genome sequencing was performed on an Illumina NovaSEQ 6000 platform (San Diego, CA). The raw data quality control was conducted using fastQC software version 0.11.9. The reads were quality trimmed using fastp software version 0.21 [9], and the trimmed reads were used for *de novo* assembly in SPAdes software version 3.15.0 [10]. Taxonomic identification of the draft genome was conducted using the Jspecies web server based on the BLAST average nucleotide identity (ANIb) algorithm. Annotation of structural genes (*i..e.,* RNAs, CDS) was carried out using NCBI PGAP [11]. The functional gene annotation related to aromatic compound degradation was conducted using eggNOG [12] and KEGG [13] databases.

## Ethics Statements

Ethics statements is not applicable for this study.

## CRediT Author Statement

**Asmadamia Abdul Aziz:** Data curation, Formal analysis, Investigation, Writing – original draft. **Azuan Syafiq Zulbahri:** Data curation, Formal analysis, Investigation, Methodology. **Mohd Faez Sharif:** Supervision, Validation, Resources, Writing – review & editing. **Mohd Azrul Naim Mohamad:** Supervision, Conceptualization, Writing – review & editing. **Han Min Gan:** Supervision, Conceptualization, Software, Writing – review & editing. **Nur Hafizah Azizan:** Supervision, Funding acquisition, Project administration, Validation, Writing – review & editing.

## Declaration of Competing Interest

The authors declare that they have no known competing financial interests or personal relationships that could have appeared to influence the work reported in this paper.

## Data Availability

Genome sequence dataset of Bacillus Altitudinis Strain ST14 (Original Data) (NCBI). Genome sequence dataset of Bacillus Altitudinis Strain ST14 (Original Data) (NCBI). Genome sequence dataset of Bacillus Altitudinis Strain ST14 (Original Data) (NCBI). Genome sequence dataset of Bacillus Altitudinis Strain ST14 (Original Data) (NCBI).

## References

[1] Kim K., Ara S., Kabir E., Brown R.J.C. (2013). A review of airborne polycyclic aromatic hydrocarbons (PAHs) and their human health effects. Environ. Int..

[2] Srivasta M., Srivasta A., Yadav A., Rawat V. (2019). Source and control of hydrocarbon pollution. Hydrocarb. Pollut. Its. Eff. Environ..

[3] Ebakota O.D., Osarueme J.O., Gift O.N., Odoligie I., Osazee J.O. (2017). Isolation and characterization of hydrocarbon-degrading bacteria in top and subsoil of selected mechanic workshops in Benin City Metropolis, Nigeria. J. Appl. Sci. Environ. Manag..

[4] Spini G., Spina F., Poli A., Blieux A.L., Reignier T., Gramellini C., Varese G.C., Puglisi E. (2018). Molecular and microbiological insights on the enrichment procedures for the isolation of petroleum degrading bacteria and fungi. Front. Microbiol..

[5] Lima S.D., Oliviera A.F., Golin R., Lopes V.C.P., Caixeta D.S., Lima Z.M., Morais E.B. (2020). Isolation and characterization of hydrocarbon-degrading bacteria from gas station leaking-contaminated groundwater in the Southern Amazon, Brazil, Brazilian. J. Biol..

[6] Alkaabi N., Al-Ghouti M.A., Jaoua S., Zouari N. (2020). Potential for native hydrocarbon-degrading bacteria to remediate highly weathered oil-polluted soils in Qatar through self-purification and bioaugmentation in bio-piles. Biotechnol. Rep..

[7] Borah D., Yadav R.N.S. (2014). Biodegradation of complex hydrocarbon by a novel *Bacillus cereus* strain. J. Environ. Sci. Technol..

[8] Ab Wahab S.U.K., Hairudin H.F., Aris M.S.M., Chowdhury A.J.K. (2021). Analysis of water quality parameters using environmetric technique for health assessment of rivers pollution sources pre COVID-19 pandemic in Gebeng Industrial Area, Pahang, Malaysia. Malaysian J. Med. Health Sci..

[9] Chen S., Zhou Y., Chen Y., Gu J. (2018). Fastp: an ultra-fast all-in-one FASTQ preprocessor. Bioinformatics.

[10] Bankevich A., Nurk S., Antipov D., Gurevich A.A., Dvorkin M., Kulikov A.S., Lesin V.M., Nikolenko S.I., Pham S., Prjibelski A.D., Psyhkin A.V., Sirotkin A.V., Vyahhi N., tesler G., Alekseyev M.A., Pevzner P.A. (2012). SPAdes: a new genome assembly algorithm and its applications to single-cell sequencing. J. Comput. Biol..

[11] Pruitt K.D., Tatusova T., Klimke W., Maglott D.R. (2012). NCBI reference sequences: current status, policy, and new initiatives. Nucleic Acids Res.

[12] Cantalapiedra C.P., Hernández-Plaza A., Letunic I., Bork P., Huerta-Cepas J. (2021). eggNOG-mapper v2: functional annotation, orthology assignments, and domain prediction at the metagenomic scale. Mol. Biol. Evol..

[13] Kanehisa M., Araki M., Goto S., Hattori M., Hirakawa M., Itoh M., Katayama T., Kawashima S., Okuda S., Tokimatsu T., Yamanishi Y. (2008). KEGG for linking genomes to life and the environment. Nucleic Acids Res.

